# A prospective observational study for a Federated Artificial Intelligence solution for moniToring mental Health status after cancer treatment (FAITH): study protocol

**DOI:** 10.1186/s12888-022-04446-5

**Published:** 2022-12-21

**Authors:** Raquel Lemos, Sofia Areias-Marques, Pedro Ferreira, Philip O’Brien, María Eugenia Beltrán-Jaunsarás, Gabriela Ribeiro, Miguel Martín, María del Monte-Millán, Sara López-Tarruella, Tatiana Massarrah, Fernando Luís-Ferreira, Giuseppe Frau, Stefanos Venios, Gary McManus, Albino J. Oliveira-Maia

**Affiliations:** 1 Champalimaud Research & Clinical Centre, Champalimaud Foundation, Lisbon, Portugal; 2 ISPA – Instituto Universitário de Ciências Psicológicas, Sociais E da Vida, Lisbon, Portugal; 3 Department of Electrical and Computer Engineering, Faculdade de Ciências E Tecnologia, Universidade Nova de Lisboa, Lisbon, Portugal; 4 Waterford Institute of Technology, Waterford, Ireland; 5 LifeSTech, Department of Photonics and Bioengineering, Escuela Técnica Superior de Ingenieros de Telecomunicación, Universidad Politécnica de Madrid, Madrid, Spain; 6 NOVA Medical School, Faculdade de Ciências Médicas, NMS, FCM, Universidade NOVA de Lisboa, Lisbon, Portugal; 7 Medical Oncology Department, Hospital General Universitario Gregorio Marañón, IiSGM, CIBERONC, Geicam, Universidad Complutense, Madrid, Spain; 8 Medical Oncology Department, Hospital General Universitario Gregorio Marañón, IiSGM, CIBERONC, Madrid, Spain; 9 Deep Blue, Rome, Italy; 10 Suite5 Data Intelligence Solutions Limited, Limassol, Cyprus

**Keywords:** Cancer, Depression, Survivorship, Federated learning, Artificial intelligence, Wearables, Remote assessment, Quality of life

## Abstract

**Background:**

Depression is a common condition among cancer patients, across several points in the disease trajectory. Although presenting higher prevalence rates than the general population, it is often not reported or remains unnoticed. Moreover, somatic symptoms of depression are common in the oncological context and should not be dismissed as a general symptom of cancer. It becomes even more challenging to track psychological distress in the period after the treatment, where connection with the healthcare system typically becomes sporadic. The main goal of the FAITH project is to remotely identify and predict depressive symptoms in cancer survivors, based on a federated machine learning (ML) approach, towards optimization of privacy.

**Methods:**

FAITH will remotely analyse depression markers, predicting their negative trends. These markers will be treated in distinct categories, namely nutrition, sleep, activity and voice, assessed in part through wearable technologies. The study will include 300 patients who have had a previous diagnosis of breast or lung cancer and will be recruited 1 to 5 years after the end of primary cancer. The study will be organized as a 12-month longitudinal prospective observational cohort study, with monthly assessments to evaluate depression symptoms and quality of life among cancer survivors. The primary endpoint is the severity of depressive symptoms as measured by the Hamilton Depression Rating Scale (Ham-D) at months 3, 6, 9 and 12. Secondary outcomes include self-reported anxiety and depression symptoms (HADS scale), and perceived quality of life (EORTC questionnaires), at baseline and monthly. Based on the predictive models gathered during the study, FAITH will also aim at further developing a conceptual federated learning framework, enabling to build machine learning models for the prediction and monitoring of depression without direct access to user’s personal data.

**Discussion:**

Improvements in the objectivity of psychiatric assessment are necessary. Wearable technologies can provide potential indicators of depression and anxiety and be used for biofeedback. If the FAITH application is effective, it will provide healthcare systems with a novel and innovative method to screen depressive symptoms in oncological settings.

**Trial registration:**

Trial ID: ISRCTN10423782. Date registered: 21/03/2022.

## Background

There were an estimated 3.9 million new cases of cancer in Europe in 2018 [1]. Due to advances in cancer screening and early diagnosis, cancer survival rates have increased substantially over the past decades, with approximately half of patients diagnosed with cancer expected to survive for 10 years or more [2]. Cancer survivors may face different physical and/or psychosocial sequelae caused by the illness or the treatments. Frequently, these patients report symptoms such as pain, fatigue, cognitive impairment, sexual dysfunction, sleep disturbances, distress, anxiety, or depression [3–5].

Both depression and anxiety are common symptoms among cancer patients, with higher prevalence rates compared to the general population [6]. Noteworthy, the proportion of subjects with anxiety/depression is higher among cancer survivors up to 5 years since cancer diagnosis [7], although such symptoms are often unnoticed or are dismissed as general symptoms of cancer [4, 8]. Among long‐term cancer survivors, a recent review [3] estimated that the pooled prevalence of patients with depressive symptoms was 21%. Until depression is eventually diagnosed and treated, these patients have gone through substantial suffering and experienced greatly reduced quality of life. Individuals with unaddressed and/or untreated depression are more likely to experience exacerbation of symptom burden and are less likely to exercise, present worse quality of life, greater psychological burden on the family, and increased health care utilization and expenditures [9–11]. Also, depression may be related to increased suicidality [6]. The early identification of depressive symptoms is thus of critical relevance.

Most studies focusing on psychological problems in cancer survivors have explored their prevalence mainly within the first years after diagnosis, when patients are still participating in regular follow‐ups at the hospital [12, 13]. Conversely, the prevalence of depression and/or anxiety, in cancer survivors, beyond the first years after diagnosis has receiving limited attention so far. Nevertheless, a higher prevalence of symptoms of depression among 2 years or longer survivors of breast cancer was reported when compared to the general female population [14]. Importantly, most cancer follow‐up schedules in hospitals start to decrease to 6-month or 1-year visits after the end of treatments. During this period, there is a limited connection between the patient and the healthcare environment.

Self-reporting instruments are often used in clinical practice to detect depressive symptoms and assess their severity [15]. They have the advantage of being quick, easy to administer, and inexpensive, but rely on psychological and cognitive symptoms rather than other manifestations of depressive disorders. Nevertheless, somatic symptoms such as fatigue, loss of appetite and sleep disturbances, are relevant when diagnosing depression in the context of cancer, and therefore, they should also be carefully considered [16]. Importantly, self-rated and symptom severity scales used in screening settings are not adequate to make a clinical diagnosis of depression, that requires a structured or semi-structured clinical interview. Somatic symptoms, such as trouble falling or staying asleep or sleeping too much, feeling tired or having little energy, poor appetite or overeating and trouble concentrating, have been reported to have higher accuracy for diagnosis of depression among patients with cancer, with insomnia as the optimal symptom for case-finding and screening properties [16]. Changes in appetite were also reported as the most pronounced somatic symptom in patients with depression and cancer [17, 18]. Importantly, while somatic symptoms of depression are common in patients with cancer, even when they are not depressed, they should nevertheless be considered as they are even more common in the context of depression [16].

Nutrition, appetite, and weight regulation play a relevant role both in cancer and depression [19, 20]. There are bidirectional associations between depression symptomatology, appetite, and weight dysregulation [20, 21]. Patients with depression may experience decreased or increased appetite, a phenomenon which is poorly understood [21], including in cancer survivors. Sleep disturbances are also common in patients with cancer, with reports of 30% to 60% experiencing insomnia [22], which may persist for years after cancer treatments [23]. Importantly, in the general population, insomnia was shown not only to be associated with depression but also to be an independent risk factor for its development [24]. Furthermore, a strong association between sleep disruption and depression in patients with cancer has been reported [25, 26]. Fatigue or loss of energy are greatly associated both with cancer and depression. In fact, it was revealed to be the second most frequently reported criterion for depression among those of the Diagnostic and Statistical Manual of Mental Disorders (DSM) classification, experienced by 87.9% of patients with major depressive disorder (MDD) [27] beyond the oncological context. Individuals presenting fatigue or loss of energy are more likely to engage in lower levels of physical activity (PA). Indeed, people with depression have previously been shown to be less physically active and more deconditioned than nondepressed individuals [28]. Interestingly, a growing number of studies assessing the benefits of PA in depression, confirm it as an effective intervention [29]. Moreover, at a preventive level, robust evidence has supported a protective role of PA on the risk for developing MDD [30, 31]. Finally, speech patterns have been known to provide indicators of mental disorders. Reports go back to 1921, with Kraepelin describing that the voice of patients with depression tended to have lower pitch, more monotonous speech, lower sound intensity, and lower speech rate, as well as more hesitations, stuttering, and whispering [32]. Importantly, research has shown voice/speech analysis to be a strong predictor of depression severity [33, 34] and of antidepressant treatment responses [35, 36]. In fact, speech presents several advantages in the context of depression: its content is a direct way of expressing emotions, while motor and acoustic variation is an indirect measure of neural modulation generalizable across languages [37].

The assessment of depression is usually performed through symptom-based questions, typically within instruments that can be general somatic scales or more general measurements of psychopathology. Ideally, however, features of somatic symptoms would be measured along with the associated phenomena. Thus, the development of an objective, non-invasive, physiologically based marker that captures depression severity would provide new avenues for clinical research and most directly for development of diagnostic instruments in the context of mental health. Moreover, nowadays it is becoming easier to collect data on patterns of voice or activity using smartphones, tablets, and computers. Therefore, technology using data collected in the clinic and/or remotely could represent a promising possibility towards improving depression diagnosis, and thus become a reliable biomarker.

Information and Communication Technologies (ICT) has provided innovations that can assist patients with chronic illness, such as depression. This ranges from the use of devices that can communicate different types of physiological and behavioural data, remotely evaluated by a health care expert, to mobile applications (apps) designed for intervention for specific health problems. Improvements in the objectivity of psychiatric assessments have been promised through health‐related data collection using sensors (e.g., wearables, smartphones, cameras) alongside the use of Machine Learning (ML) methodologies [37]. Wearables, including watches, rings, and clothes that measure biological and behavioural metrics, such as temperature, movement and heart rate, can be potential indicators of anxiety and depression, and used to provide biofeedback [38]. Additionally, smartphones can provide important measurements, including voice and activity data, to detect mood disorders like depression [39]. Nevertheless, most healthcare data is hard to obtain due to legal, privacy, technical, and data-ownership challenges, especially among international institutions [40]. An emerging solution to this problem is Federated Learning (FL), a new approach to ML where the training data does not leave the users’ local device. FL has shown that a deep learning model could be trained with 99% of the accuracy of the same model obtain through traditional data-sharing methods [41]. In the future, ML models are thus expected to be trained without the need of computing resources owned by giant Artificial Intelligence (AI) companies, with users not being required to compromise privacy for improvement of services.

The main goal of the FAITH project is to remotely analyse depression markers, predicting their trends, in cancer survivors. These depression markers will be treated under several distinct categories: Nutrition, Sleep, Activity, and Voice. Central to the FAITH project is the introduction of only one new device – a *smartband*, which, together with the already existing smartphone functionality, is able to cast a wide enough net over a user’s health data. The FAITH application (app) will be designed to essentially run in the background, and with the least user input as possible. Based on the predictive models developed during the study, FAITH also aims at developing a conceptual federated learning framework, enabling it to build ML models for the prediction and monitoring of depressive symptoms without direct access to user’s personal data. The use of the optimized model might further allow early screening and proper intervention, thus leading towards early improvements of quality of life.

The primary objective of the FAITH project is to remotely identify and predict the risk or trends of depression in cancer survivors. The project will use the FAITH app and Point of Care solution (Alpha Version) that collects information from a smartphone, smartband and questionnaires in breast and lung cancer survivors. The FAITH model will include variables such as sociodemographic, clinical, psychosocial factors, and depression markers (Nutrition, Sleep, Activity, and Voice).

The secondary objectives are to determine the effectiveness of several bio-related variables in:

- Measuring longitudinal progression of established depression markers and of quality of life and assessing their relationship.
- Examining the potential differences in the predictive and outcome variables according to cancer subtype (breast or lung cancer).
- Developing a conceptual federated learning depression predictive model that will provide the healthcare staff with advanced warnings, allowing for timely intervention.
- Evaluating FAITH´s solution feasibility and usability in cancer care.

## Methods

### Outcomes

The primary outcome will be severity of depressive symptoms as measured by the Hamilton Depression Rating Scale (Ham-D_17_), a semi-structured interview, at months 3, 6, 9 and 12 post-baseline. Secondary outcomes include self-reported anxiety and distress according to the HADS scale, and perceived quality of life measured by EORTC questionnaires at baseline and monthly until month 12.

### Study design

The study is a multi-centre longitudinal prospective observational cohort study. Patients will be recruited at two clinical centres: Hospital General Universitario Gregorio Marañón (HGUGM), in Spain and Champalimaud Clinical Centre, at the Champalimaud Foundation (CF), in Portugal.

**Fig. 1:**
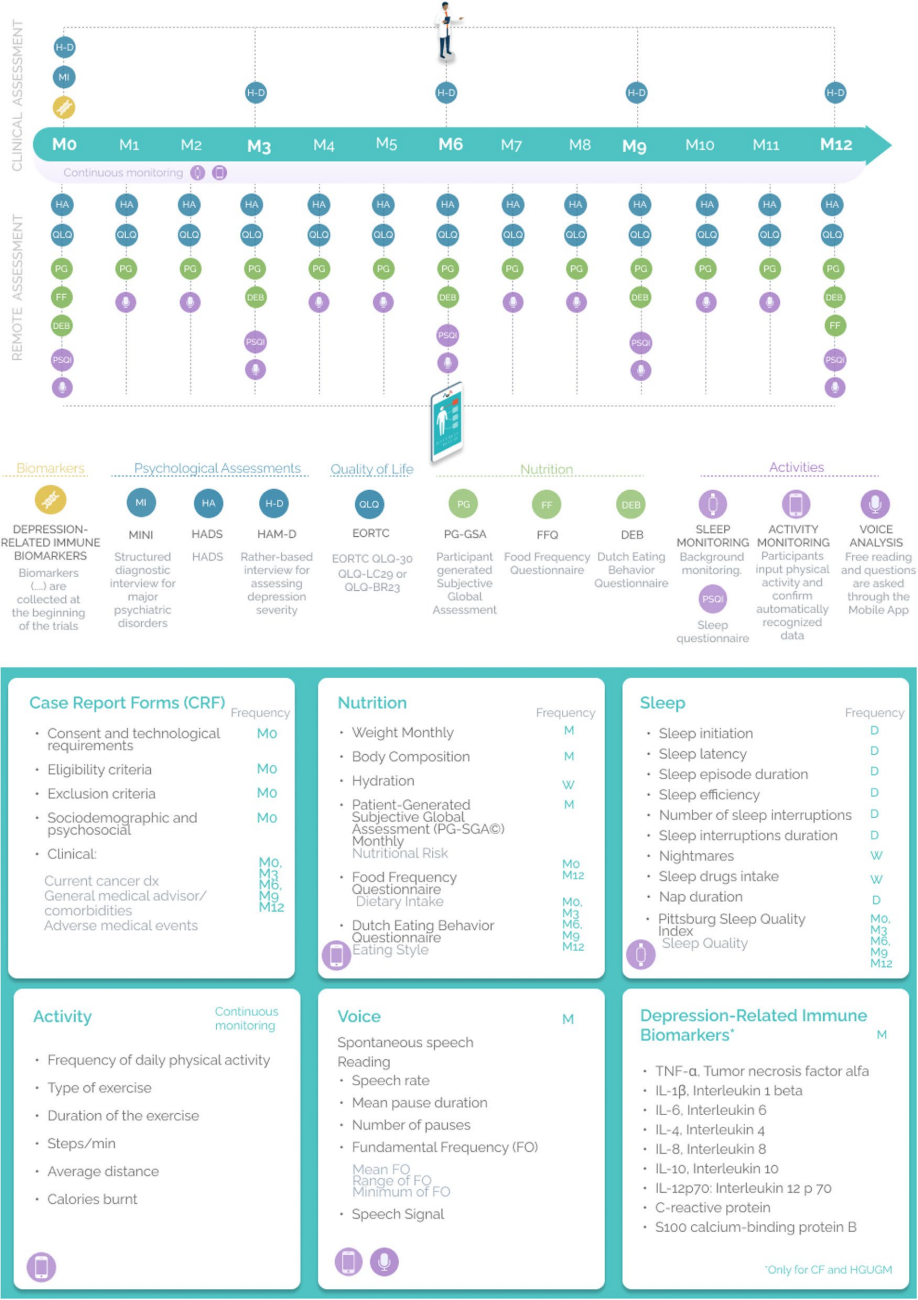
FAITH study design

Each pilot site will identify patients ambispectively, by inspection of medical records collected in each clinical centre, aiming to select breast and/or lung cancer survivors according to the defined inclusion criteria. The study is designed as a 12-month longitudinal study, with monthly (± 1 week) assessments, to evaluate depression symptoms and quality of life in cancer survivors (Fig. 1). Upon informed consent signature and eligibility screening, patients will perform baseline assessments and will receive the study IT tools (smartband and the smartphone app) and the respective instructions. Patients will provide monthly assessments through the FAITH mobile app regarding the self-rated questionnaires (HADS and EORTC QLQ global and specific modules). Every 3-months patients will be contacted by phone by a clinical researcher to be monitored for Depression severity using the Ham-D_17_ interview (Fig. 1). Other variables of interest (Nutrition, Sleep, Activity, and Voice) will be monitored continuously through the FAITH mobile app and smartband (Fig. 1). During the study, participants will be notified about missing data by automated alerts to optimize protocol compliance.

### Study population

The target sample is 300 cancer survivors, in total. HGUGM will recruit breast cancer patients, and CF will recruit both breast and lung cancer patients. Patients will be referred to the study by their oncologists or contacted by a research team member. Cancer survivors are defined [42] as persons who have completed primary treatments for cancer, and are apparently free of recurrent or persistent cancer. More specifically, we will consider the period after the end of primary treatment, when there is a lower risk of recurrence [43]: [1-5] years after the end of primary cancer treatments.

#### Inclusion criteria

Eligible participants include both breast and lung cancer patients, that should meet the following criteria:

- Capable of giving signed informed consent
- Age between 18–70 years
- Native or fluent Spanish/Portuguese speakers
- Be Apple iPhone or Android users
- For Apple iPhone users: have iOS 14 + installed on the device
- For Android device users: have Android 7 + and Google Fit installed on the device
- Histologically confirmed invasive breast cancer or invasive lung cancer
- Breast cancer participants must have stage I to III at diagnosis and histopathology of Hormonal receptor (HR) positive / Human epidermal growth factor receptor 2 (HER2) negative; HR positive /HER2 positive or HR negative /HER2 positive disease
- Lung cancer participants must have stage I to IIIA/B at diagnosis
- Must have received (neo) adjuvant chemotherapy +/− biologicals, definitive surgery, and/or adjuvant radiotherapy as standard of care, within at least 1 year, and up to 5 years, from the last curative intent treatment
- Patients under ongoing hormone therapy, chemotherapy or other disease-specific treatments will be included when these treatments are conducted to reduce the risk of relapse
- Performance status (ECOG): 0–2

#### Exclusion criteria

A lack of capability to participate in the study including giving consent, illiteracy or otherwise not understanding the study’s instructions will result in ineligibility. Individuals who demonstrate an inability to use smartphone technology, especially touch screen interaction and basic maintenance procedures of the devices (e.g. charging, switching on and off, reading notifications, making calls, and sending and receiving text messages) will also be considered ineligible. Patients’ ability to use smartphone technology will be assessed by asking whether they are able to use smartphones to check and send emails.

Clinical exclusion criteria include:

- Distant metastases
- Inflammatory breast cancer
- Small cell lung carcinoma
- A previous invasive malignancy whose treatment was completed within 5 years before the diagnosis of the current neoplasic disease, apart from adequately treated, basal or squamous cell skin carcinoma or curatively resected cervical cancer *in situ*
- Any clinically significant acute medical illness or other active and insufficiently controlled concomitant disease, such as the following examples for cardiac disease: congestive heart failure, symptomatic coronary artery disease or cardiac arrhythmia not well controlled with medication, or myocardial infarction within the last 12 months
- Major surgery for a severe disease or trauma which could affect patient’s psychosocial wellbeing (for example, major heart or abdominal surgery) within 4 weeks prior to study entry or lack of complete recovery from the effects of surgery
- Any treatment for major illnesses in the last 6 months that will preclude the compliance to this protocol as per the investigator discretion
- Pregnancy or breastfeeding at time of recruitment
- A diagnosis of a current moderate to severe major depressive episode at baseline according to the M.I.N.I. (version 7.0.2) (diagnosis) and/or Ham-D_17_ (severity)
- A current or previous hypomanic or manic episode, current or previous psychotic disorder or current mood disorder with psychotic symptoms, as well as substance abuse or dependence in the last 12 months, as reported or screened by the M.I.N.I.
- Presence of any psychiatric disorder requiring urgent care or hospitalization at the time of recruitment
- Presence of neurologic disorder or any previously known major structural lesion of the central nervous system
- Developmental disorders with low intelligence quotient or any other form of cognitive impairment

### Materials

During the study, several materials will be used to collect the main variables of interest, namely depression and quality of life. The presence of a psychiatric diagnosis according to DSM-5 criteria will also be evaluated (M.I.N.I.) by a trained clinical researcher.

The rater-based and self-report scales and interviews that will be administered at baseline and at each follow-up evaluation include:

- Mini-International Neuropsychiatric Interview (M.I.N.I. 7.0.2) [44] – is a short structured diagnostic interview for the major psychiatric disorders of DSM-5. It will only be done at the screening visit to verify psychiatric eligibility criteria.
- Hamilton Rating Scale for Depression (Ham-D_17_) [45] – rater-based interview for assessing depression severity. The Ham-D will be done at baseline to ensure screening eligibility, and every 3-month by a phone interview.
- Hospital Anxiety and Depression Scale (HADS) [46] – patient self-rated digital version to monitor levels of depression and anxiety. The HADS will be done monthly in the FAITH mobile app.
- EORTC QLQ-30 (European Organization for Research and Treatment of Cancer quality-of-life questionnaire) [47]: the QLQ-30 will be done monthly in the FAITH mobile app. Subscale modules will be used according to cancer subtype: the EORTC QLQ-BR23 Breast Cancer [48] and the EORTC QLQ-LC29 Lung Cancer [49] at the same time-points.

Clinical data will be obtained from electronic health records or other clinical registries in ambispective fashion.

### FAITH platform

**Fig. 2:**
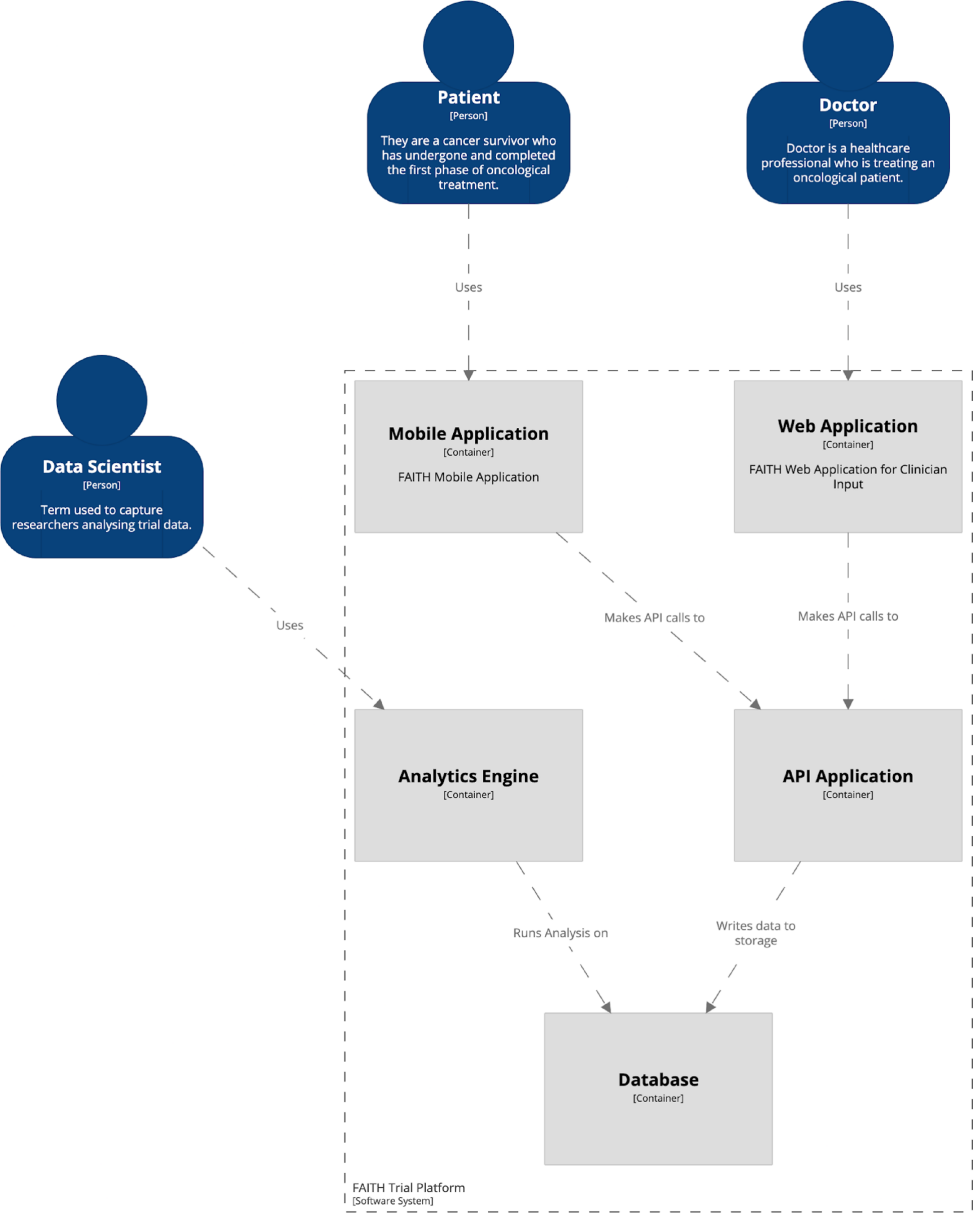
FAITH Trial Platform Overview

The FAITH platform is hosted in a private cloud through Amazon Web Services (AWS) which ensures no public access to the cloud and enhanced security access for sensitive data. Thus, only staff from the two hospitals working in FAITH, as well as the data scientists from the consortium will have access to the aggregated and coded data. The platform (Fig. 2) comprises four layers with the following components:

- Application Layer:
  - ◦ Mobile app to capture the variables of interest from the smartphone and the smartband.
  - ◦ Web app to record the Case Report Form (CRF) data.
  - ◦ An analytics engine to ultimately run queries against the recorded data (to do statistical analysis and generate the future FAITH Model).
- The services Layer:
  - ◦ An Application Programming Interface (API) to serve as an endpoint for both the mobile and web applications.
  - ◦ A security access and authorization access service and Audit Logs for complete visibility into who is accessing what data.
  - ◦ The connection services to register and access data from databases.
  - ◦ The statistical analytic tools to enable the analytic engine to further analyse data.
- The data Layer:
  - ◦ A database to store the recorded data from the trial and the app.
- The security Layer:
  - ◦ A Transversal layer to the other three layers and guarantees that information is stored and transferred between them all, ensuring that data follows GDPR rules and is secure.

### FAITH app

The FAITH mobile app packages the functionality from the four constituent modules: nutrition, sleep, activity, and voice. It is built using React Native to ensure compatibility with both iOS and Android devices. The Nutrition module is implemented through the integration of validated questionnaires in the mobile app. This allows for a streamlined capture of reliable data. The voice analysis will be implemented by capturing the user’s voice directly in the app, and then offloading this recording. Since the methods used in the literature vary, it has been chosen an approach that is expected to perform optimally in the overall context of the FAITH study: 1) Patients will respond to a set of defined questions (to elicit spontaneous speech) and read a text (e.g., *The Grandfather Passage* 1); 2) The questions will follow a form [35] with the frequency reference updated appropriately.^2^ Participants will use the FAITH app on their smartphone for all voice recordings, at monthly intervals.

### Wearables

FAITH leverages the suite of sensors available in modern smartphones and augments it with a complimentary device for activity and sleep tracking (Fitbit). Together, these two devices provide enough data to track the key depression markers, without requiring users to adopt multiple devices. Sleep tracking is achieved through a combination of Google Fit and the Fitbit. The participant must wear the Fitbit and install its associated App (Fitbit app) to collect data from the device and hand it to Google Fit, intrinsic to most Android devices. In the case of IOS devices, data will be transmitted via the Fitbit App to Apple health. The App will transmit information necessary for the characterisation of sleep. Ambient light will be measured in Lux, using the smartphone’s light sensors within the FAITH App, to improve the quality of sleep assessment. This will be an additional input to help determining sleep latency and sleep duration with precision. The FAITH app will also collect activity data, from a Fitbit, by means of the Activity Monitor Module (AMM). Along with the remaining modules, the AAM will be integrated within the app to seamlessly capture data without intervention from users. Instead, it will leverage on GoogleFit and Apple HealthKit for Android and iOS devices, respectively. Data coming from both GoogleFit and Apple HealthKit (or calculated in the activity tracker itself) will be stored locally on the device following the FHIR compatible data model.

### Data storage

The data will be stored in Amazon Relational Database Service (RDS) for PostgreSQL, an open-source object-relational database. Amazon RDS makes it easy to set up, operate, and scale PostgreSQL deployments in the cloud. The automated backup feature of Amazon RDS enables recovery of the PostgreSQL database instance to any point in time within the specified retention period up to thirty-five days. The data is secured through a security layer (access, authorizations, cybersecurity, etc.), applied over the pseudonymization approach.

### Statistical analyses

Descriptive statistics will be generated for all clinical characteristics and outcome measures as appropriate.

In FAITH, variables will be selected using algorithms based on different feature selection methods. For example, for wrapper methods, we will use forward selection, backward elimination, and stepwise selection. These methods can also support filtering, as they evaluate possible combinations of features against the evaluation criterion [50]. The evaluation criterion depends on the type of problem e.g. for regression, evaluation criteria can be *p*-values, R-squared, Adjusted R-squared. Similarly, for classification, the evaluation criteria can be accuracy, which in this case will work as filtering. These methods will help towards eliminating unnecessary variables, or substitute variables with others that will yield a similar prediction [50].

This step of variable selection is fundamental to determine the set of variables that will provide the best fit for generating a model. The correct interaction of variables will support the identification of correlations and interplays among clinical, sociodemographic, psychographic and depression markers, gaining deeper insights for more effective models.

Pre-selected statistical tools according to suitability to support modelling of the selected markers as predictors of depression comprise temporal data mining, time-series analysis, sequence classification methods, clustering of time series, temporal pattern discovery and association rules. If any of these techniques is found not to be suitable, a similar alternative technique will be explored. Because the FAITH data is non-stationary, time series forecasting and neural network analysis modelling will be used to predict future values, for example of presence and severity of depression, based on observed and data of the pre-selected markers. Thus, this statistical methodology will support forecasting by creating a predictive model for risk of depression, anxiety, and quality of life. Additionally, there are several time-series forecasting algorithms that may be useful for this analysis, among which Autoregressive and Moving Average to predict future values as a combination (e.g., linear combination) of values obtained from the sample.

While missing data could introduce bias, and potentially even invalidate results and conclusions, excluding participants with missing data could affect the power of analysis. The strategy of the FAITH project to handle missing data within its prospective datasets will be at first to identify a model for the missing data, which can be based on its distribution conditioned by the available data, as well as to detect if it can be categorized by whether the missing data is random or not. Secondly, missing data patterns will be checked as to detect if only one variable is missing (univariate) or more than one variable (multivariate, monotone, and non-monotone). According to this analysis, the most suitable method to address missing values will be selected among the following: replacing missing values with mean of observed values, omitting participants with missing values (complete case analysis), missing indicator method (creating dummy variable, e.g., zero as an indicator for missing data), mean substitution (replacing missing value with overall mean or subgroup mean). More complex methods may also be considered, such as maximum likelihood method or multiple imputation.

Although FL is a central component of this project, it is important to clarify that it will only be implemented after the trial is concluded (beta version), and not throughout its duration (alpha version). Likewise, an appropriate FL model can only be developed once the data is analysed.

#### Sample size

Recruitment will be based on the calculation of sample size, performed with a set of multiple linear regressions, calculated using three inputs: the expected effect size, the chosen significance level (between 0.5 – 0.10), and power (0.8 – 0.95). Based on these, we propose the following sample plan:

- An estimation to have a sample size of *n* = 231 (300 subjects, considering an estimated attrition rate of 23%) is sufficient to ensure 85% power at *p* < 0.05 for detecting the cumulative contribution of up to 27 independent predictors (from our group of variables collected from the 4 modules). Furthermore, in a regression model with 27 independent variables this sample size is sufficient to detect the significant added value of each individual independent variable assuming a small effect size (Cohen’s f2 > 0.039).

### Ethics compliance

The study was already approved by the Ethical Committees (ECs) of the participating institutions.

**Fig. 3:**
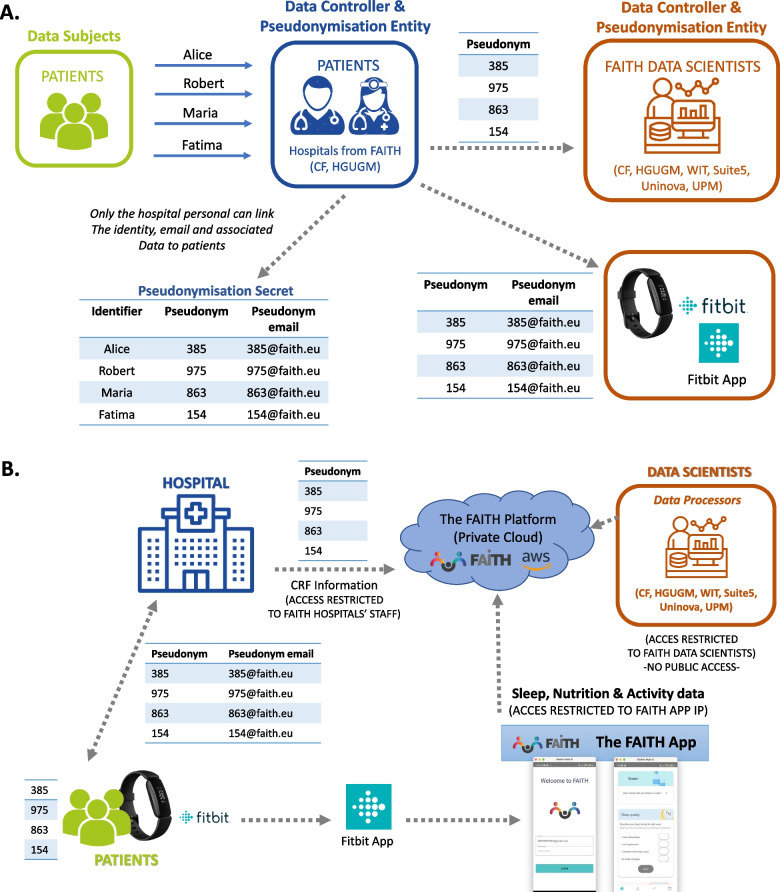
FAITH dataflow and security compliance. (Legend: Panel **A** – Pseudonymization process; Panel **B** – Flow of pseudonymized data)

FAITH uses pseudonymization as a security measure (art. 32 GDPR) in the context of data protection by design (art. 25 GDPR), hiding the identity of the participants from any third party and enabling the data protection goal of unlinkability. Each hospital will perform the pseudonymisation process. Figure 3A shows the flow of the pseudonymization process.

Taking this process into consideration, the coded data flows among the three main stakeholders: the participants, the hospitals, and the data processors (data analysts and devices), supported by the platform and the security of the platform (Fig. 3B).

The responsible Principal Investigator (PI) will ensure that this study is conducted in compliance with the protocol, adhering to the principles of Good Clinical Practice (GCP) and with current local legislation. This trial will be initiated only after all required legal documentation has been reviewed and approved by the responsible independent ethics committee (IEC) and competent authority (CA) of the hospitals according to all applying national and international regulations.

Prior to participation in the trial, written informed consent must be obtained from each participant according to ICH-GCP and to the regulatory and legal requirements of the participating country.

The application of structured and self-rated psychiatric and psychological questionnaires is exclusively for research purposes. Nevertheless, these questionnaires are clinically validated and thus have the capability of identifying clinically significant medical issues such as severe depression or suicidality. Consequently, an alarm will be sent to the research team of the corresponding hospital whenever a participant scores above pre-defined thresholds on these parameters (according to each questionnaire scoring and norming scales). The participants may then be contacted (according to the incidental findings policy) and a clinical follow-up will be proposed according to the specific internal protocol of each hospital.

Information regarding procedures in case of incidental findings is detailed in the informed consent forms. In the presence of a clinically relevant incidental finding, the participant will be notified by a study physician in accordance with consent obtained regarding incidental finding. In the absence of consent to communicate incidental findings, the issue might be brought to the local Ethics Committee for recommendations regarding actions to take. The participant will also be provided with written information about the incidental finding.

The security and privacy of participants in the FAITH project will comply with relevant EU legislation regarding processing of personal data and the protection of privacy in the electronic communications sector, Directive 95/46/EC on protection of individuals regarding the processing of personal data and on the free movement of such data, and international texts on privacy. The provisions of Regulation EU 2016/679 will be adopted, or national regulations in matters of personal data protection and privacy if they are more restrictive.

Once it has been authorized by all ECs, any protocol modification will be documented by the coordinator in the form of an amendment. All the protocol amendments must be notified to the ECs involved in the study before their application. Any amendment affecting the participant requires their informed consent prior to implementation.

## Discussion

FAITH is a multi-centre longitudinal prospective observational cohort study that will include breast and lung cancer survivors across two clinical centres (in Spain and Portugal). The purpose of including cancer survivors is to remotely assess patients when they have already totally or partially disconnected from the more specialized healthcare system. The lack of connection between the patient and the healthcare environment may lead to psychological changes not being reported and remaining unnoticed. This protocol has been designed to implement the FAITH study, aiming to remotely analyse depression markers and predict onset of depression among cancer survivors. The depression markers include domains with important roles both in cancer and in depression: nutrition, sleep, activity, and voice. Presence or absence of symptoms of depression will be assessed regularly through standardized Depression instruments both self-rated (HADS) and rater-based (Ham-D). Measures of quality of life will also be obtained.

In FAITH, the remote assessment of patients will be done through the patient’s smartphone, synced with a smartband through the FAITH App. This functionality will allow the extraction of the depression markers of interest. The FAITH application will be designed to run in the background, with the least user input as possible. Based on the predictive models developed during the study, FAITH also aims at developing a conceptual federated learning framework, enabling it to build ML models for monitoring depressive symptoms without direct access to user’s personal data. FAITH is expected to provide a federated learning model to predict depression, that will enable healthcare providers to offer advanced warnings, allowing for timely mental health intervention in oncological settings. The optimized FAITH model, through early screening and intervention, would be a relevant clinical tool for the improvement of quality of life.

## Data Availability

Not applicable.

## Abbreviations

AMM: Activity Monitor Module
API: Application Programming Interface
AWS: Amazon Web Services
AC: Competent Authority
CF: Champalimaud Foundation
CRF: Case report form
DSM: Diagnostic and Statistical Manual of Mental Disorders
EORTC QLQ: European Organization for Research and Treatment of Cancer quality-of-life
ECOG: Eastern Cooperative Oncology Group
ECs: Ethical Committees
FHIR: Fast Healthcare Interoperability Resources
HADS: Hospital Anxiety and Depression Scale
Ham-D: Hamilton Rating Scale for Depression
HER2: Human epidermal growth factor receptor 2
HGUGM: Hospital General Universitario Gregorio Marañón
HR: Hormone receptor
ICH-GCP: International Conference on Harmonisation-Good Clinical Practice
GDPR: General Data Protection Regulation
FAITH: Federated Artificial Intelligence solution for moniToring mental Health status
MDD: Major Depressive Disorder
M.I.N.I.: Mini-International Neuropsychiatric Interview
ML: Machine Learning
ORR: Overall response rate
PA: Physical Activity
PI: Principal Investigator
RDS: Amazon Relational Database Service

## Footnotes

1
https://www.amyspeechlanguagetherapy.com/uploads/7/5/7/4/7574967/grandfatherpassage.pdf

2 “Describe how you’ve been feeling emotionally during the last week. Record your experiences as well as any comments other people made about your emotions.”, “Describe how you’ve been feeling physically during the past week. If you can, give examples of how your physical feelings have been affecting your life.” “Please describe how your emotional and physical feelings have affected your general ability to function in the past week. Consider such things as your ability to work, manage your home, get along with others, and participate in leisure activities.”.
